# The current state of palliative care research in heart failure

**DOI:** 10.1093/eschf/xvag067

**Published:** 2026-03-02

**Authors:** Charlotte Gross, Ankit Gupta, Ruth Burgess, Lucy Ziegler, Everlien De Graaf, Tiny Jaarsma, Klaus K Witte, Sam Straw

**Affiliations:** Leeds Institute of Cardiovascular and Metabolic Medicine, LIGHT Building, University of Leeds, Clarendon Way, Leeds LS2 9JT, UK; Leeds Institute of Cardiovascular and Metabolic Medicine, LIGHT Building, University of Leeds, Clarendon Way, Leeds LS2 9JT, UK; Leeds Institute of Cardiovascular and Metabolic Medicine, LIGHT Building, University of Leeds, Clarendon Way, Leeds LS2 9JT, UK; Leeds Institute of Cardiovascular and Metabolic Medicine, LIGHT Building, University of Leeds, Clarendon Way, Leeds LS2 9JT, UK; Julius Center, University Medical Center, Utrecht, Netherlands; Julius Center, University Medical Center, Utrecht, Netherlands; Department of Health, Medicine and Caring Sciences, Linköping University, Linköping, Sweden; Leeds Institute of Cardiovascular and Metabolic Medicine, LIGHT Building, University of Leeds, Clarendon Way, Leeds LS2 9JT, UK; Leeds Institute of Cardiovascular and Metabolic Medicine, LIGHT Building, University of Leeds, Clarendon Way, Leeds LS2 9JT, UK

**Keywords:** Supportive care, Palliative care, End of life care, Heart failure

## Palliative care and heart failure

Heart failure is the common endpoint for all cardiovascular disease processes, and despite advancements in its management is characterized by persistent symptoms, frequent hospitalization, and premature death.^1^ Palliative care is recommended as a key component of heart failure care, which aims to address the physical, psychological, social, and spiritual needs of patients and caregivers.^2^ Palliative care has been underrepresented in heart failure research—a previous study found that between 2009 and 2013, it rarely featured in cardiovascular conference proceedings or received research funding awards.^3^ Conference proceedings and clinical trial registries may help to identify current research priorities, as the former showcase the latest science whilst the latter provide a contemporaneous snapshot of the research landscape. In this *Viewpoint,* we present data describing the current state of palliative care research for heart failure, the impact of repeated calls to improve palliative care delivery, and its more prominent position in clinical practice guidelines that have occurred in the interim.

## Search strategy

We searched the conference proceedings and congress archives for six major international cardiology meetings between January 2018 and November 2023, which were:

- The American College of Cardiology (ACC) Scientific Sessions
- The Association of Cardiovascular Nursing and Allied Professions (ACNAP) Congresses
- The American Heart Association (AHA) Scientific Sessions
- The European Society of Cardiology (ESC) Congresses
- The Heart Failure Association (HFA) of the ESC Congresses
- The Heart Failure Society of America (HFSA) Annual Scientific Meetings

We manually screened ACC, ACNAP, AHA, and HFSA conference proceedings. For ESC and HFA Congresses, we developed a search strategy using the ESC365 platform due to the archive size. Search terms included ‘palliative’, ‘palliation’, ‘advanced care planning’, ‘end of life’, ‘terminal heart failure’, ‘end-stage heart failure’, and ‘last phase of life’. Search results were consolidated by removing duplicates and using session title results to locate associated abstracts and presentations; session titles were then excluded as standalone records. Where proceedings were unavailable online, we made requests to the relevant organizing committees. Outputs that focused on services, referrals, interventions, and treatments aimed at improving patients’ or caregivers’ experiences in the context of life-limiting illnesses were included. We excluded outputs relating to congenital heart disease ‘palliative’ procedures that aimed to act as a bridge to future definitive treatment. Outputs were coded as abstracts or presentations (talks or symposia), and session data were recorded and grouped. We categorized outputs as palliative care or palliative care for heart failure; the latter was assigned when heart failure was the primary patient population. We then searched major trial registration sites for studies related to palliative care for heart failure, which were:

- ClinicalTrials.gov
- The European Union Clinical Trials Register
- The ISRCTN

For consistency, the same search terms were applied. We included all completed, recruiting or not yet recruiting observational studies and clinical trials that focused on services, referrals, interventions, and treatments aimed at improving patients’ or caregivers’ experiences, in which the primary population comprised patients with heart failure. The trial year was defined by the trial start date. Data are presented as absolute numbers and proportions of total symposia presentations, abstracts, trials, and cardiovascular trials. Proportions of palliative outputs from general (ACC, ACNAP, AHA, and ESC) and heart failure specialist (HFA and HFSA) meetings and trends over time were analysed using chi-squared tests.

## International cardiology meetings

**Figure 1:**
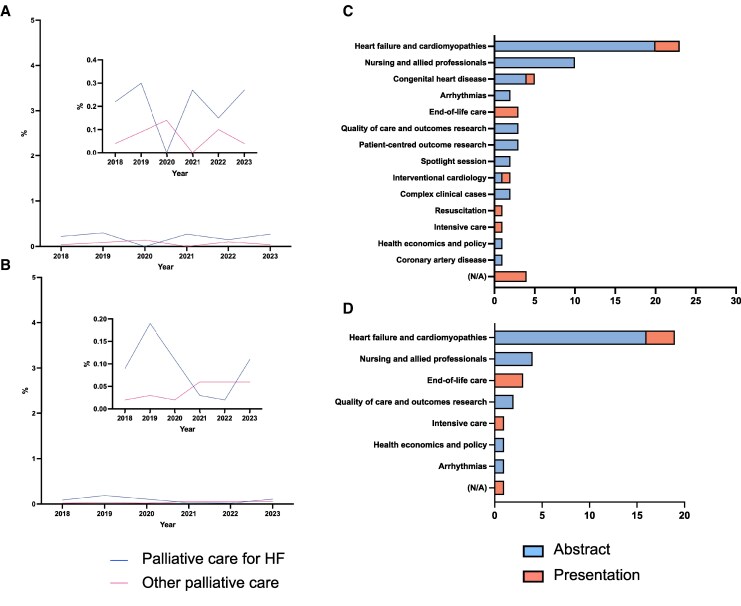
Plots of the percentage of *A* presentations and *B* abstracts at international cardiology congresses related to palliative care and palliative care for heart failure. Bar charts showing the number of presentations and abstracts for *C* palliative care and *D* palliative care for heart failure divided by session title grouping at international cardiology congresses

Our search retrieved 123 558 results. After result consolidation, 11 666 symposia or session presentations and 67 288 abstracts across the 32 congresses were identified. Of these, 39 (0.33%) presentations and 91 (0.14%) abstracts concerned palliative care, and 25 (0.21%) presentations and 60 (0.09%) abstracts focused specifically on palliative care for heart failure. Proportions of palliative care and palliative care for heart failure symposia presentations did not differ across years (*P* = .879 and *P* = .687) while proportions of abstracts varied year-on-year with no clear trend (*P* = .076 and *P* < .001) (*Figure 1*).

Presentations and abstracts from general cardiology meetings most commonly appeared in ‘heart failure and cardiomyopathies’ sessions. Among these, 10 (40%) had no specific sub-session focus. ‘End-of-life’ was the second most common sub-category within this grouping: 6 abstracts (24%) in total, 5 (21%) from one session at AHA Scientific Sessions 2019. Outside ‘heart failure and cardiomyopathies’ sessions, only 6 presentations and no abstracts were categorized as ‘end-of-life’ across all general cardiology meetings. At HFA congresses, 15 (45%) presentations were presented in the session grouping ‘palliative and end-of-life care’. HFSA session titles were structural, e.g. ‘abstracts’ and ‘clinical care’, which limited comparability to other society meetings. Heart failure specialist meetings had a higher proportion of presentations and abstracts on palliative care (0.62% vs 0.24%, *P* = .002; 0.38% vs 0.09%, *P* < .001) and palliative care for heart failure (0.59% vs 0.09%, *P* < .001; 0.33% vs 0.05%, *P* < .001) compared to the general cardiology conferences.

## Clinical trial registries

In our search of registries, we found no relevant studies in the EU Clinical Trials Register or the ISRCTN. ClinicalTrials.gov had 15 completed, 2 recruiting, and 3 withdrawn or terminated clinical trials for which palliative care for heart failure was the focus. The earliest included trial began in 2011. Palliative care for heart failure focused trials represented 0.059% of cardiovascular and 0.008% of all completed or recruiting clinical trial entries within ClinicalTrials.gov between 01/01/2010 and 31/12/2023. The proportions of clinical trials focused on palliative care for heart failure were different across years, but with no clear trend (*P* = .040).

## A greater focus on palliative care has not resulted in more research activity

Despite repeated calls for the earlier integration of palliative care into the heart failure management pathway, position statements, and greater prominence in the ESC and AHA/ACC/HFSA heart failure guidelines,^4–8^ we found that palliative care seldom featured in conference proceedings or clinical trial registries. Since 2008, international guidelines have placed a greater emphasis on the importance of quality of life and symptom control.^4^ For example, the 2009 HFA position statement and the 2016 ACC expert consensus offer a framework for integrating holistic palliative care into the heart failure management pathway.^5,6^ The 2021 ESC and 2022 AHA/ACC/HFSA guidelines recommend the early integration of palliative care and the implementation of a multidisciplinary approach.^7,8^ This marks a clear evolution in prioritization, though in this analysis, we found no evidence of any impact on research activity. Our data show consistently low numbers of presentations at international conferences, which may limit the visibility of palliative care within the cardiovascular community. While representation was higher at heart failure specialist meetings, the presentation frequency remained low, with very few ongoing clinical studies.

## The unmet palliative care needs of people living with heart failure

Over 64 million people are currently living with heart failure worldwide, and all cardiovascular disease processes ultimately progress to heart failure.^9^ Despite, or potentially because of advances in guideline-directed medical and device therapies, more patients are living with heart failure and its associated symptom burden and impaired quality of life.^10^ Palliative and supportive care remains inconsistently integrated into routine heart failure management, and underutilized compared with other diseases such as cancer.^11^ This is despite clear evidence from the PALlitative care in Heart Failure (PAL-HF) trial, which showed an interdisciplinary multicomponent palliative care intervention improved quality of life, depression, anxiety, and spiritual wellbeing.^12^ Other benefits include improved patient satisfaction and reduced symptom burden, rehospitalization, and mechanical ventilation.^13^ However, evidence is limited on implementing palliative heart failure care pathways and identifying who benefits—data from the Palliative Care intervention in Heart Failure (PCHF) trial suggests little benefit when patients are targeted indiscriminately.^14^

## Potential solutions

Several structural factors mean this evidence gap persists. Prognostic uncertainty and the complexity of palliative care interventions in heart failure create methodological and ethical challenges in trial and study design. Fragmented pathways across cardiology, primary care, and specialist palliative care services limit consistent identification and follow-up. Clinicians would benefit from clear implementation strategies for palliative care, rather than broad recommendations, although this is challenging without evidence to guide best practice. Using a ‘dual-track framework’ for heart failure management, which offers guideline-directed medical therapy with concurrent palliative care, might optimize therapy while contingency planning for potential deterioration. The EU-Horizons consortium project ‘Raphael’ will provide information on the benefits of integrating needs-assessed palliative care into heart failure services on quality of life, survival, health economics, and optimal delivery.^15^ In our study, palliative care outputs mainly featured under broad session titles, rather than dedicated palliative or end-of-life sessions, which might raise the profile of this research.

## Limitations

Requests for total conference outputs from ESC, HFSA, and AHA meetings were unsuccessful, although manual EndNote™ counts are accurate to the best of our knowledge. Second, symposia presentation data were unavailable for ACC and HFSA meetings, limiting comparisons between general and heart failure specialist conferences. Third, the ESC keyword search may have missed relevant outputs, though this is unlikely given the ESC365 Boolean modifiers. Finally, archive availability varied, affecting year-to-year comparisons.

## Conclusions

Repeated calls for the integration of palliative care into the heart failure pathway, as well as dedicated position statements and a greater prominence in heart failure guidelines, have not translated into greater research activity.

## Declarations

### Data Availability

The data generated for this analysis are available from the corresponding author upon reasonable request.
